# Delamination of the Annulus Fibrosus of the Intervertebral Disc: Using a Bovine Tail Model to Examine Effect of Separation Rate

**DOI:** 10.3389/fbioe.2022.883268

**Published:** 2022-06-28

**Authors:** K. Josh Briar, John G. McMorran, Diane E. Gregory

**Affiliations:** ^1^ Department of Kinesiology and Physical Education, Wilfrid Laurier University, Waterloo, ON, Canada; ^2^ Department of Health Sciences, Wilfrid Laurier University, Waterloo, ON, Canada

**Keywords:** annulus fibrosus, interlamellar matrix, viscoelasticity, peel strength, peel strength variability, intervertebral disc

## Abstract

The intervertebral disc (IVD) is a complex structure, and recent evidence suggests that separations or delamination between layers of the annulus may contribute to degeneration development, a common cause of low back pain The purpose of the present experiment was to quantify the mechanical response of the layer-adjoining interlamellar matrix at different rates of separation. Understanding the rate-dependency of the interlamellar matrix, or the adhesion between adjacent layers of the disc, is important as the spine experiences various loading velocities during activities of daily living. Twelve discs were dissected from four bovine tails (three extracts per tail). Two multi-layered annulus samples were collected from each IVD (total = 24, mean bond width = 3.82 ± 0.96 mm) and randomly assigned to a 180° peel test at one of three delamination rates; 0.05 mm/s, 0.5 mm/s, or 5 mm/s. Annulus extracts were found to have similar maximal adhesion strengths (*p* = 0.39) and stiffness (*p* = 0.97) across all rate conditions. However, a significant difference in lamellar adhesion strength variability was observed between the 5 mm/s condition (0.96 N/mm ± 0.31) when compared to the 0.5 mm/s (0.50 N/mm ± 0.19) and 0.05 mm/s (0.37 N/mm ± 0.13) conditions (*p* < 0.05). Increased variability may be indicative of non-uniform strength due to inconsistent adhesion throughout the interlamellar matrix, which is exacerbated by increased rates of loading. The observed non-uniform strength could possibly lead to a scenario more favourable to the development of microtrauma, and eventual delamination.

## Introduction

Low back pain is the leading cause of disability worldwide (Vos et al., 2015; Vos et al., 2017). The origin of intervertebral disc (IVD) related back pain is largely unknown, but research suggests that separations of annular layers (delamination) marks a significant biomechanical compromise of the annulus fibrosus’ (AF) structure (Iatridis and Ap Gwynn, 2004; Gregory et al., 2014; Tavakoli et al., 2018). The majority of the adhesive interlamellar matrix (ILM) bonding adjacent lamellae is composed of type VI collagen, proteoglycans, water, and elastic fibres (Melrose et al., 2008; Tavakoli et al., 2016). Collagen fibres provide strength to the ILM to hold adjacent lamella together, a role shared by the elastic fibres which also contribute an elastic behaviour to this matrix (Melrose et al., 2008; Tavakoli et al., 2016). These elastic fibres have been reported to span the ILM space, lending radial support across adjacent layers (Schollum et al., 2009; Schollum et al., 2018), creating the trans-lamellar bridging network (TBLN) which acts to transmit forces radially through the AF (Schollum et al., 2008; Yu et al., 2015). The proteoglycans within the ILM may provide a role in force-dispersion through mechanisms of slippage between adjacent fibrils (Cribb and Scott, 1995). The ILM’s composition of elastic fibres and proteoglycans imparts this structure with viscoelastic mechanical properties (Cohen et al., 1976; Tavakoli and Costi, 2018a; Tavakoli and Costi, 2018b). The interaction between the ILM and the lamellae of the AF have been shown to contribute to the tensile capacity to the AF, as demonstrated through previous shear strain research on these structures (Fujita et al., 2000).

As such, it appears plausible that the ILM’s material behaviour will differ according to the rate at which it undergoes stress, suggesting that the mechanical characteristics of delamination may not be a uniform process across different velocities of loading (Tavakoli and Costi, 2018b). Due to the high variability of daily movement, the ILM is subsequently subject to loading at different rates. Increased variability in lamellar adhesion strength may represent a greater probability that a mismatch between the load applied to the ILM, and the ability for the ILM to bear said load, will occur across a given region of the AF resulting in delamination. Delamination of the AF involves separation of lamellae, which can result in decreased strength of this structure (Gregory et al., 2014). Previous work considering the effect of rate dependent properties of the AF have acknowledged a viscoelastic behaviour under shear testing, however, these observations were not able to isolate the ILM or its adhesive properties, especially under failure testing (Iatridis et al., 1999). Additionally, the effect of delamination rate on adhesion strength variability has not been previously reported. Interestingly, defects in adhesion of various non-biological composites have been shown to result in variability in the adhesion strength (Rodríguez-Ramos et al., 2013). Similarly, a weakened and/or compromised AF may also increase the variability of adhesion strength which could in turn increase the possibility of circumferential tearing that can lead to IVD herniation, spinal nerve root compression, and a significant state of degeneration and pain in the spine (Iyer and Kim, 2016; Calvo-Echenique et al., 2018).

There is a paucity of research describing the rate-dependent mechanical behaviour of the ILM during delamination. Therefore, the purpose of the present experiment was to examine the mechanical response of AF tissue extracts to different rates of experimental delamination. Due to the viscoelastic components of the ILM, it was hypothesized that increasing rates of delamination would result in greater measures of lamellar adhesion strength, lamellar adhesion strength variability, and lamellar adhesion stiffness in AF extracts.

## Methodology

### Specimens

**FIGURE 1:**
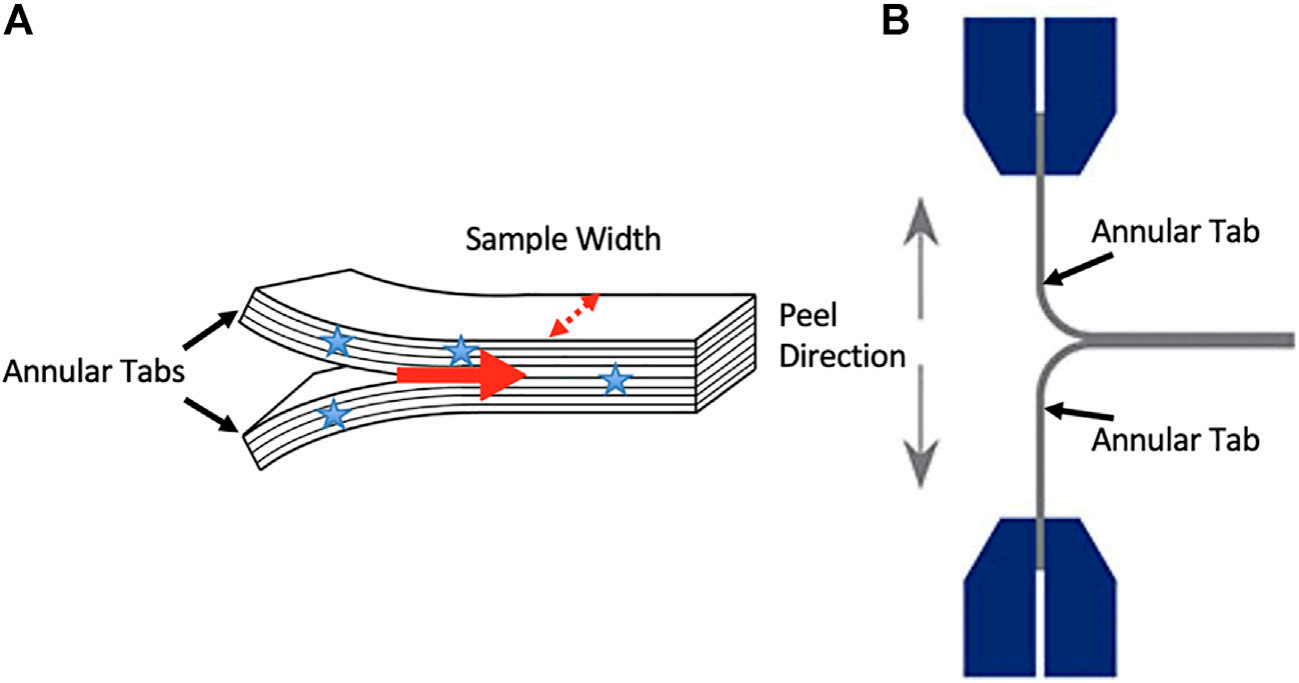
**(A)** An illustration of the annular tabs created in specimens with stars indicating where sample width measurements occurred. The red arrow indicates the direction of delamination propagation. An illustration of a sample mounted in the testing system and the orientation of the peel direction **(B)**.

Twelve intervertebral discs (IVDs) were obtained from the tails of 4 skeletally mature bovine spines. The spines were obtained from a common source which allowed for control over variables such as physical activity levels, diet, and age (approximately 18 months). Specimens were stored at −20° until testing. Prior to testing, each sample was removed from the freezer and thawed at room temperature for 16 h. The bovine tail typically has a substantial amount of muscle tissue and as a result, 16 h was needed to fully thaw the discs under the muscle. Attempts with shorter thawing periods resulted in frozen regions of the disc at the time of dissection. Two multilayer samples were removed from the lateral portion of each IVD (*n* = 24), and each of these multilayer samples was manually delaminated with a scalpel. The separation of two central, adjacent layers, created T-shaped tabs (approximately 1 cm in length), with their conjoining ILM (approximately 4 cm in length) isolated between them (Figure 1A). Each sample’s bond width was measured with digital calipers (average bond width 3.82 ± 0.97 mm). This process involved taking four measurements along the juncture where the 1 cm tabs met and the mean of these four measurements indicated sample bond width. During dissection, samples were kept moist with phosphate buffered saline (PBS).

### Testing Protocol

The multilayer tissue samples were mounted into a tensile testing system (UStretch, Cellscale, Waterloo, ON, Canada) via the manually created tabs in order to conduct a lamellar adhesion test with a 180^o^ lamellar adhesion-test configuration (Gregory et al., 2012); Figure 1B. Just prior to testing, samples were misted with PBS. The samples were randomly separated into three delamination rate groups (*n* = 8 per group); 0.05 mm/s (slow), 0.5 mm/s (medium), 5 mm/s (fast). Force and displacement data were sampled at 30Hz during each lamellar adhesion test and force measures were normalized by dividing by tissue bond width.

### Analysis Protocol

**FIGURE 2:**
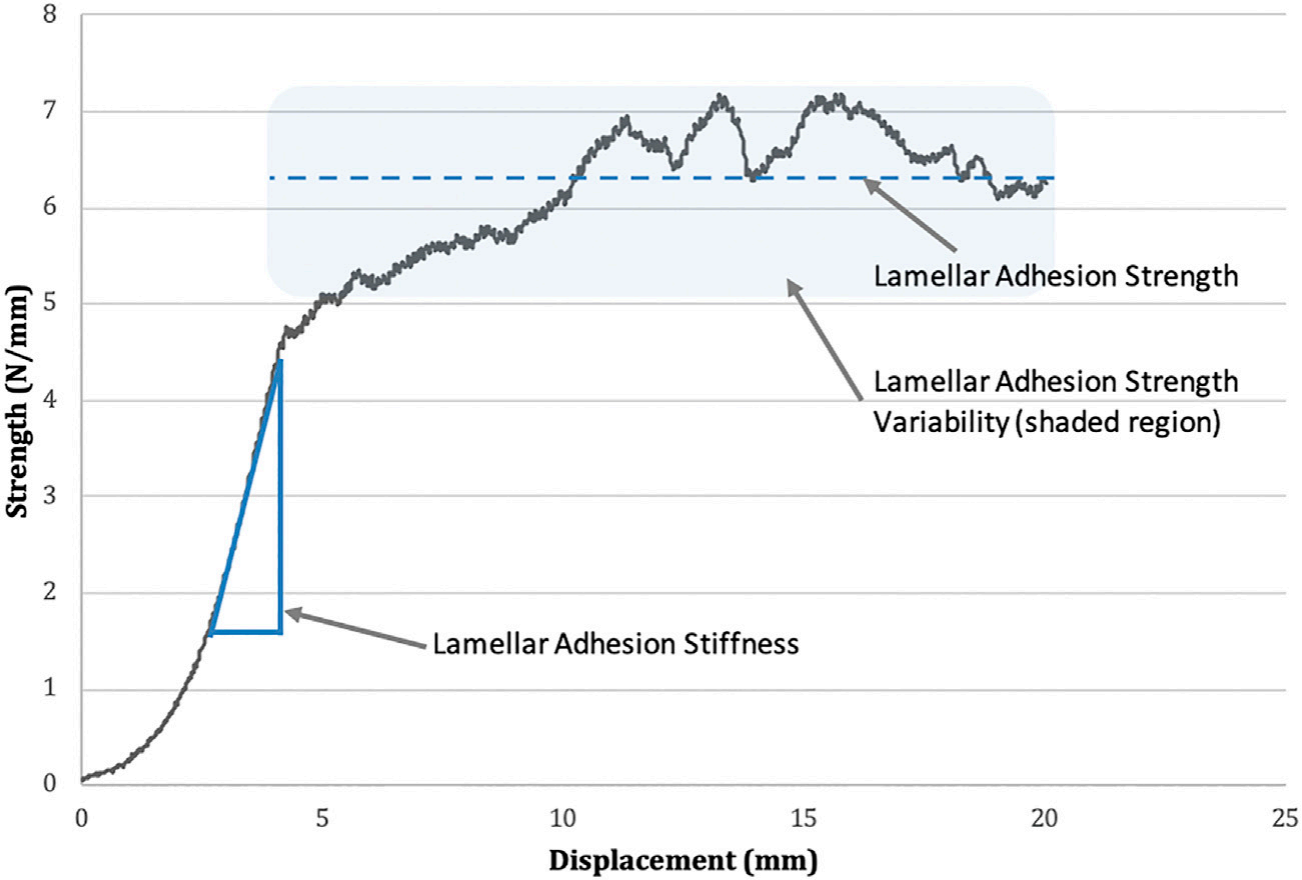
Lamellar adhesion strength-displacement curve. Mean lamellar adhesion strength is represented by the dotted line. The standard deviation of lamellar adhesion strength is represented by the shaded area above and below the line of the mean (termed lamellar adhesion variability). Lamellar adhesion stiffness is indicated as the slope of the linear region of the graph.

The variables of interest from the normalized adhesion strength-displacement curves (Figure 2) were as follows: (A) average lamellar adhesion strength (N/mm): this was calculated as the average strength (N/mm) over the plateau region of force displacement curves, divided by tissue bond-width (mm); (B) lamellar adhesion strength variability (N/mm); this was calculated as the standard deviation of lamellar adhesion strength across the plateau region; (C) lamellar adhesion stiffness (N/mm^2^): this was calculated as the slope of the linear region of the lamellar adhesion strength-displacement curve (R^2^ > 0.95). The plateau region was defined as the region on the normalized adhesion strength-displacement curves following the first point of inflection where the average strength value remained relatively stable.

### Histology

Two additional unloaded bovine annulus samples were dissected and fixed in 10% formalin for 48 h prior to histological processing at the Ontario Veterinary College (University of Guelph, Guelph, Canada). The IVD was removed from the sample by cutting along the endplate on both the inferior and superior faces. In order to fit into the histology cassettes, discs were then cut in half along the sagittal plane yielding a left and right side of the disc. Half discs were then placed in the cassettes such that sections were cut in the axial plane in order to image the layers radially from the inner AF to the outer AF. Samples were then embedded in paraffin and sectioned and stained with Safranin O/ Fast Green Stain. Slides were imaged with an Axiolab microscope (Zeiss, Jena, Germany); bright field, non-polarizing; at ×10 magnification.

### Statistical Analysis

A one-way analysis of variance (ANOVA) was performed on the data to determine if there was a statistical difference in lamellar adhesion strength, lamellar adhesion strength variability, or lamellar adhesion stiffness, based upon the rate at which the lamellar adhesion test was performed. All IVD samples were treated as independent samples due to similar variance observed within and between animals. For statistical analysis, a Shapiro-Wilks test was used to determine normality and an alpha level of 0.05 was utilized for this analysis. A post-hoc Tukey’s Studentized Range Test was performed to determine differences between groups when a main effect was found.

## Results

One sample within the 5 mm/s group exceeded the limits of the tensile system load cell (45N) and was not included in the final results as all other sampled failed at loads under 25N. This sample was omitted as it deemed there was error in tissue mounting.

### Lamellar Adhesion Strength and Stiffness

There was no main effect of rate on the lamellar adhesion strength of the AF (F = 1.00, *p* = 0.38). Specifically, the mean strength (standard deviation) for the 0.05 mm/s test group was 3.43 N/mm (1.34), 4.51 N/mm (2.07) for the 0.5 mm/s test group, and 3.87 N/mm (0.89) for the 5.0 mm/s test group. There was also no main effect of experimental conditions on the lamellar adhesion stiffness of the AF (F = 0.03, *p* = 0.97). Lamellar adhesion stiffness was found to be 0.65 N/mm^2^ (0.72) for the 0.05 mm/s test group, 0.66 N/mm^2^ (0.37) for the 0.5 mm/s test group, and 0.60 N/mm^2^ (0.24) for the 5.0 mm/s test group.

### Lamellar Adhesion Strength Variability

**FIGURE 3:**
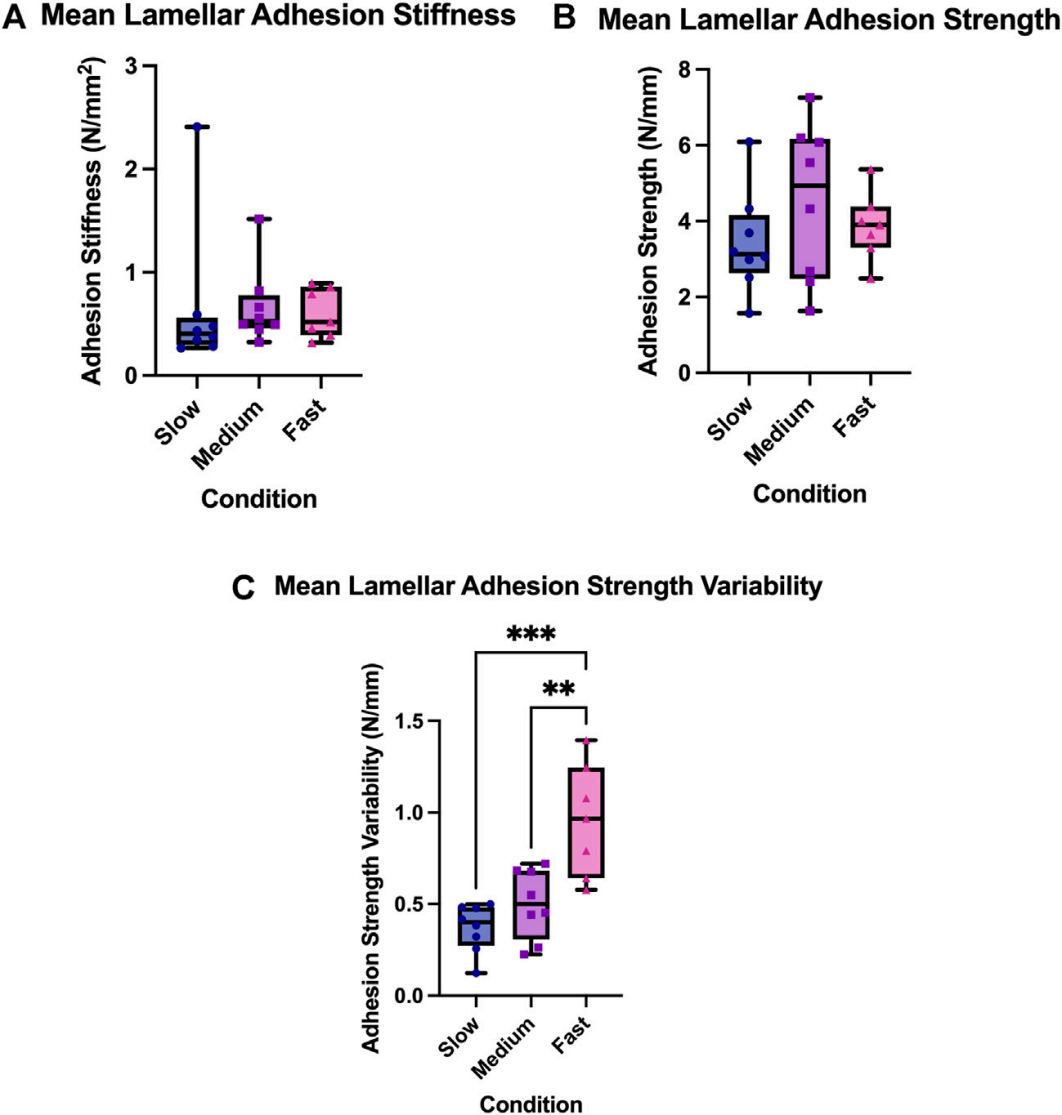
**(A)** Lamellar adhesion variability, **(B)** Lamellar adhesion strength, and **(C)** Lamellar adhesion strength variability for the three experimental rate condition; the slow condition (0.05 mm/s), the medium condition (0.5 mm/s), and the fast condition (5.0 mm/s). The box extends from the 25% percentile to the 75% percentile value, the line inside the box represents the median, and the error bars indicate the range for each experimental group. Asterisks indicate significant differences between groups (*p* < 0.05).

**FIGURE 4:**
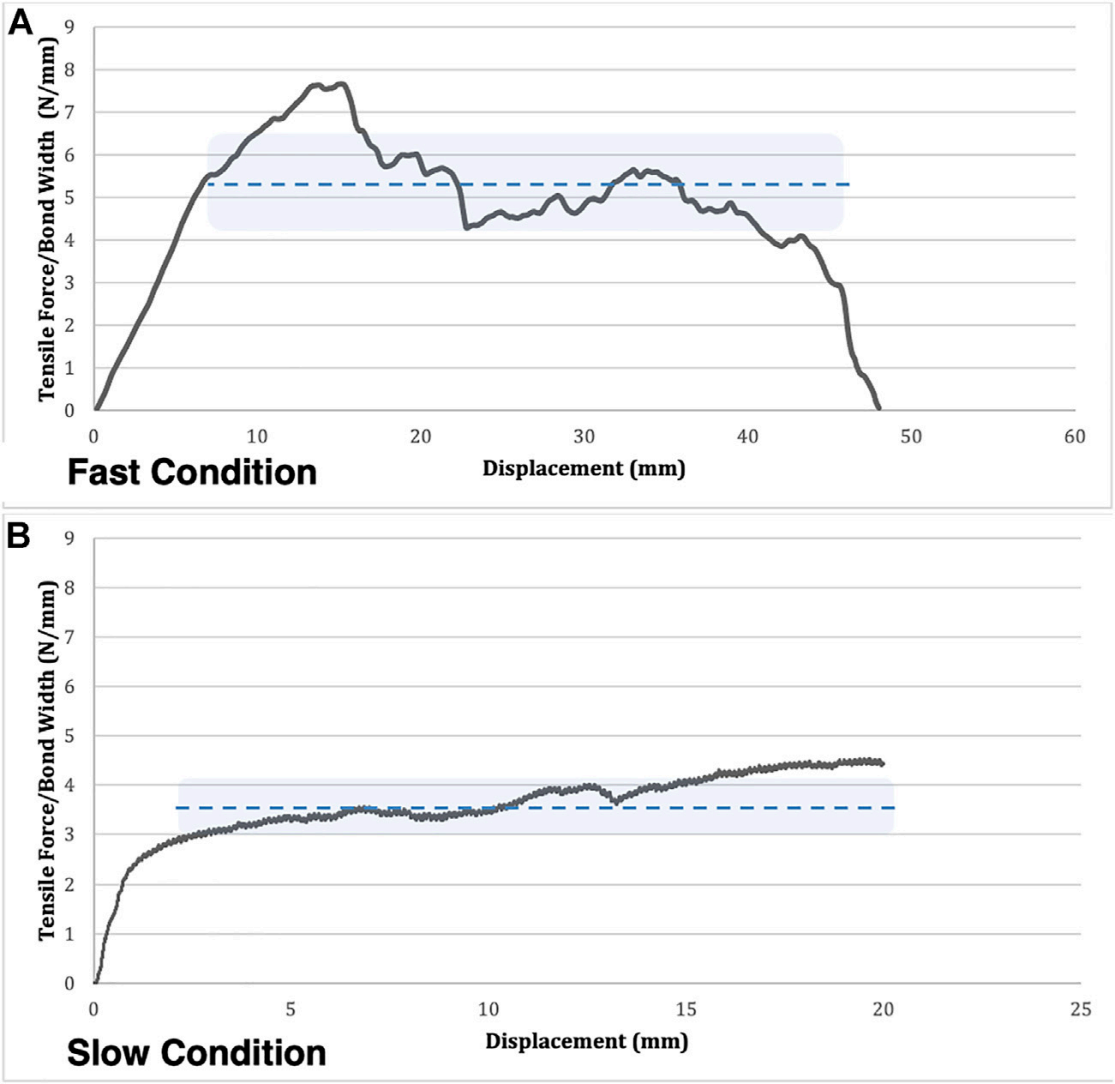
A comparison of a typical adhesion-strength curve for a fast **(A)** and slow **(B)** condition trial. Mean lamellar adhesion strength is represented by the dotted line. The lamellar adhesion strength variability is represented by the shaded area above and below the line of the mean. Note the higher variability across the plateau in the fast condition compared to the slow condition.

There was a main effect of experimental conditions on lamellar adhesion strength variability (F = 14.92, *p* = 0.0001); Figures 3, 4. Specifically, mean adhesion strength variability was found to be 0.37 N/mm (0.13) for the 0.05 mm/s test group, 0.50 N/mm (0.19) for the 0.5 mm/s test group, and 0.96 N/mm (0.31) for the 5.0 mm/s test group. Tukey’s post hoc analyses determined that there was a significant difference between the 5.0 mm/s and 0.5 mm/s group (CI = [0.17,0.74]), and between the 5.0 mm/s and 0.05 mm/s group (CI = [0.30,0.87]) such that as rate of delamination increased, lamellar adhesion strength variability also increased.

### Histology

**FIGURE 5:**
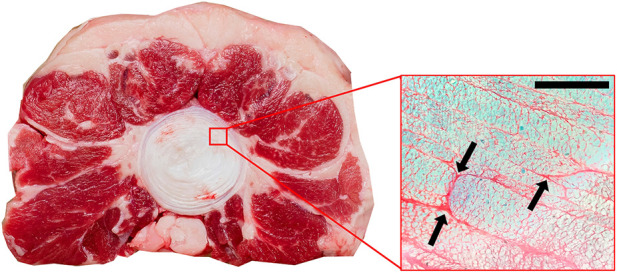
Axial view of annulus fibrosus stained with Safranin O/Fast Green (×10 magnification). Arrows indicate regions of inconsistent adhesion between adjacent lamellae. These regions are generally where one collagen bundle ends and an adjacent one begins. Scale bar 500 µm.

Histological analysis indicated regions of inconsistent adhesion between the layers of the annulus. These areas generally included regions of incomplete lamellae (Figure 5A) or where one lamellae terminated and the next successive lamellae began (Figure 5B).

## Discussion

The purpose of the present experiment was to explore the influence of delamination rate on the adhesive properties of the ILM. While no differences in lamellar adhesion strength or stiffness were observed, the current study did find that lamellar adhesion strength variability was affected by rate. Specifically, this work observed increased strength variability with increasing rate of delamination. Variability in the bond strength has been attributed to imperfections in adhesion in various non-biologic composite materials (Rodríguez-Ramos et al., 2013). Previous work performed in other non-biological adhesive materials, for example, cellophane (Gardon, 1963; Campbell and Flake, 2004) as well as biologic tissue samples including the degenerated AF (Gregory et al., 2014) and cartilage (Bürgin et al., 2014), have noted non-uniform bond strength and have attributed these observations to variations in stress distributions across the bond as well as in the material composition of the bond itself. Bond strength variability during the peel tests in the current work was also likely and indication of variations and/or inconsistencies in the matrix composition between the AF layers.

Physiologically, these variations are important as regions of low adhesion strength are more susceptible to delamination which could increase risk of damage to the AF as a whole. Previous work in a rabbit model has shown that degenerated IVDs have lower adhesion strength compared to non-degenerated IVDs (Gregory et al., 2014). In the current work, when delamination velocity was low, the variations in matrix composition were not perceptible and the result was relatively stable peel strength over the length of the bonded samples; however, when higher rates of loading were examined, peel strength fluctuated in magnitude, likely due to viscoelastic nature of the variations in material composition (i.e. the TLBN (Schollum et al., 2008; Yu et al., 2015)). It is at these higher rates where regions of low delamination strength may pose a risk of injury to the IVD.

No significant differences in lamellar adhesion strength or stiffness were observed between the different delamination rates, aligning with some previous work done conducted in degenerated human IVDs (Gregory et al., 2012). In contrast, other research has shown that the matrix that spans the interlamellar space behaves viscoelastically in response to strain during dynamic loading (Tavakoli and Costi, 2018a; Tavakoli and Costi, 2018b); however these studies examined the ILM under different load orientations (i.e. radial tension and shear) than what was conducted in the current study. The peel test in the current study aims to quantify the tensile strain in the ILM when resisting delamination (Gregory et al., 2012).

A limited number of studies have reported adhesion strength and variability in the AF including in the bovine tail (Harvey-Burgess and Gregory, 2019), rabbit (Gregory et al., 2014), porcine (Snow et al., 2018; Ghelani et al., 2020; McMorran and Gregory, 2021), and human (Calvo-Echenique et al., 2018) IVD. The peel strength values reported in the current study are similar to those reported previously in the bovine tail and porcine; while the reported values in the rabbit and human IVD were much lower (less than 1.0N/mm); however this may be a result of the high degree of degeneration in those experiments. The studies conducted in the bovine tail and porcine IVD also reported peel strength variability which was found to be similar to those reported in the current work.

It is important to discuss the current study’s limitations. First, bovine caudal IVDs were used, which have distinct anatomy, structure, and loading conditions compared to the human lumbar IVDs. It must be noted that bovine tails are non-weight bearing however, animal studies have reported similar microstructures between bovine tail IVDs and human IVDs (Demers et al., 2004; Alini et al., 2008). While microstructures have been shown to be similar, the reported peel strengths are much higher than those reported previously in human IVDs (Gregory et al., 2012) though this may be due to the degenerated state of the human IVDs when compared to bovine making direct comparisons impractical. Future work should aim to quantify adhesion peel strength variability in the human IVD. Second, the annular samples were removed from the lateral portions of the IVD to create nearly-identical samples rather than anterior and posterior which is typically done. However, this was due to the anatomical symmetry of the bovine tail IVD. Third, observations of adhesive strength occurred over a limited range of relatively slow rates. While this observation was limited by the equipment used, future work should focus in observing the effect of strain rates like those experienced during traumatic events. The current work utilized a range of delamination rates most likely to be experienced during voluntary daily movement; however, traumatic events can exceed the rates presented in this work (greater than 5 mm/s), which would be the focus of future work. Last, given that this is the first study to examine the effect of peel test rate, future work should also aim to verify the findings by replicating the work. However, given the high variability of these data, caution should be taken when interpreting the findings of this work; reproducibility, as a result, will also likely be challenging.

## Conclusion

The present experiment did not observe a significant difference in the lamellar adhesion strength and lamellar adhesion stiffness of the ILM. In contrast, faster delamination rates were shown to influence the lamellar adhesion strength variability of the ILM, possibly highlighting a viscoelastic response to this structure. Such mechanical behaviour may permit the development of microtrauma at lower overall levels of force, and thereby present an injury scenario where the disc becomes more susceptible to delamination.

## Data Availability

The raw data supporting the conclusions of this article will be made available by the authors, without undue reservation.
